# Danggui Buxue Tang, an ancient Chinese herbal decoction, protects β-amyloid-induced cell death in cultured cortical neurons

**DOI:** 10.1186/s12906-018-2411-6

**Published:** 2019-01-08

**Authors:** Guowei Gong, Baohui Qi, Yan T. Liang, Tina T. X. Dong, Huai Y. Wang, Karl W. K. Tsim, Yuzhong Zheng

**Affiliations:** 1 Department of Biological Engineering, |Zunyi Medical University, Zhuhai Campus, Zhuhai, 519041 Guangdong China; 2 Division of Life Science and Center for Chinese Medicine, The Hong Kong University of Science and Technology, Clear Water Bay, Hong Kong, China; 3 Shenzhen Key Laboratory of Edible and Medicinal Bioresources, Shenzhen Research Institute, Shenzhen, 518000 China; 4 Department of Biology, Hanshan Normal University, Chaozhou, 521041 Guangdong China

**Keywords:** Danggui Buxue Tang, Alzhemier’s disease, Complementary and alternative medicine

## Abstract

**Background:**

Danggui Buxue Tang (DBT) is a historical Chinese herbal decoction, and which has more than 800 years of applications. This herbal decoction solely contains two materials: Astragali Radix (AR) and Angelicae Sinensis Radix (ASR) at a weight ratio of 5:1. Clinically, DBT aims to improve anemia syndrome. In complementary and alternative medicine theory, the cause of neurodegenerative disease is proposed to be related with anemia. In line to this notion, low levels of hemoglobin and red blood cell have been reported in patients suffering from Alzheimer’s disease (AD), a chronic neurodegenerative disease caused by β-amyloid peptide (Aβ) accumulation. Therefore, we would like to probe the neuroprotective functions of this ancient herbal formula in vitro.

**Method:**

The neuroprotective effects of DBT in the Aβ-induced cell death were detected in cultured cortical neurons by multiple techniques, i.e. confocal and western blot.

**Results:**

In the cultures, application of DBT reduced Aβ-induced apoptosis rate in a dose-dependent manner. In Aβ-treated cortical neurons, the expression ratio of Bcl2 to Bax was altered by DBT. In parallel, application of DBT markedly suppressed the Aβ-induced expressions of apoptotic markers, i.e. cleaved-caspase 3/9 and PARP.

**Conclusion:**

Taken these results, DBT shows promising protective effects against Aβ-induced stress or insult in cultured neurons.

## Background

Neuron is the basic unit in nervous system. It major functions are to receive incoming information and send signals to other neurons, muscles and glands. Neurons are delicate, and they are not capable of reproducing. Neurodegeneration is one type of brain diseases, resulting in progressive degeneration and/or neurons death. Alzheimer’s disease (AD), classified as one type of neurodegeneration diseases, is an irreversible disorder by smashing memory and finally destroying the performance of simple tasks [1]. The hallmark of AD diagnosis is the extracellular plaque deposition of β-amyloid peptide (Aβ) [2]. Slowing down the progression of brain deterioration in AD patients by herbal medicine is believed to be a possible treatment.

Traditional Chinese medicine has been used for thousands of years, which is also called complementary and alternative medicine. Indeed, brain problem is proposed to be related with anemia as believed in ancient theory [3, 4]. Anemia is generally caused by the dysfunction of erythropoietin (EPO) production, stimulating the proliferation and differentiation of erythroid precursor cells [5]. In line to this notion, low levels of hemoglobin and red blood cell have been reported to be correlated in AD patients [6, 7]. In parallel, Faux et al. (2014) found that low level of hemoglobin was associated with AD, as well as other cognitive impairments [8]. Astragali Radix (AR; root of *Astragalus memebranaceus* (Fisch.) Bunge var. *mongholicus* (Bunge) Hsiao) is one of herbal materials found predominantly in complementary and alternative medical decoction, and this herb is commonly used for enriching vital energy as well as stimulating EPO production [9, 10]. Astragaliside IV, one of major ingredients of AR, possesses neuroprotective effects during cerebral ischemia/reperfusion injury via anti-oxidation, anti-inflammation and anti-apoptosis [11–13]. Angelica Sinensis Radix (ASR; root of *Angelica sinensis* (Oliv.) Diel) is also called “female ginseng”: this herb protects cultured Neuro 2A cell against Aβ-triggered oxidative damage, i.e. reactive oxygen species (ROS), and which also rescues the membrane potential of mitochondrial transmembrane [14].

AR and ASR are two major herbs commonly used together to constitute herb-pair in complementary and alternative medical formulae [15, 16]. Danggui Buxue Tang (DBT), containing AR and ASR at the ratio of 5:1, is a simple herbal mixture, and this herbal mixture has been utilized for more than 800 years. DBT was recorded in ***Neiwaishang Bianhuo Lun,*** written by Li Dongyuan in AD 1247 [16, 17]. This ancient herbal formula is suggested to be consumed by patients who would like to improve the syndromes of anemia. The neuroprotective functions of AR and ASR were well demonstrated both in vitro and in vivo [18–21]. However, the neuroprotective function of DBT decoction has not been revealed yet. Hence, we are aiming to probe the neuroprotective effects of this ancient herbal decoction in cell models, as well as to reveal the underlying signaling pathway involved.

## Materials and methods

### Preparation of DBT decoction

The herbal materials of *A. memebranaceus* var. *mongholicus* (AR) and *A. sinensis* (ASR) were obtained and morphologically identified by Dr. Tina TX Dong (one of the author) in 2013. The voucher specimens of AR (# 02–10-04) and ASR (# 02–09-01) were recorded and displayed in Centre for Chinese Medicine of HKUST. To generate DBT formula, 5 parts of AR and 1 part of ASR were mixed well and boiled in water for twice, according to previous reports [16, 17]. The water extract of DBT was dried by lyophilization and re-suspended in water at 100 mg/mL final concentration. Before application, DBT was diluted to the required concentration in the cultured medium. The treatment of DBT in the Aβ-treated cultures was about 48 h, unless otherwise specified.

### Fingerprint of DBT decoction

An Agilent 1200 series system (Agilent, Santa Clara, CA) was utilized here to determine the chemical compositions of DBT decoction. The Agilent 1200 series system was equipped with degasser, binary pump, auto-sampler, and thermo-stated column compartment. Agilent, Eclipse Plus, C_18_ column (4.6 × 250 mm, 5 μm) with acetonitrile (as Solvent A) and 0.01% formic acid (as Solvent B). The detailed HLPC condition was described before [10].

### Primary cell culture

The experimental procedures were approved by The Animal Experimentation Ethics Committee of the Hong Kong University of Science and Technology (No. 17–283 for Animal Ethics Approval) and under the guidelines of “Principles of Laboratory Animal Care” (NIH publication No. DH/HA&P/8/2/3). Primary cultured cortical neurons were cultured as described previously with slightly modifications [18, 22]. Cortex was dissected by trypsin from embryonic day 18 rats by 100 mg/kg of Ketamine and 10 mg/kg of Xylazine treatment. The dissociated cortical neurons were cultured in neural basal medium with B27 and 0.5 mM GlutaMax at 37 °C incubator supplying with 5% CO_2_. Cells were cultured for 14 days before treatments. Reagents for cell cultures were purchased from Invitrogen Technologies (Carlsbad, CA).

### Cell viability assay

MTT assay was used for analysis cell viability (Sigma-Aldrich, St. Louis, MO). In brief, cells were seeded in 96-well plate and then performed drug treatment for 48 h. After that, MTT solution was added into the cultures and incubated for 2 h, the purple crystal production was dissolved by DMSO solvent and detected at 570 nm absorbance. In crystal violet assay, cells were seeded and cultured in 6-well plate. Colonies were fixed with an ice-cold methanol-acetic acid solution (3:1) for 30 min at − 20 °C and stained with 1% crystal violet for 30 min at room temperature and visualized.

### Western blot

The protein expressions of Bcl2, Bax, cleaved-PARP, cleaved-caspase 3/9 and β-actin were revealed by western blot. Cultures were seeded onto 6-well plate. After drug treatment, the cultures were harvested in high salt lysis buffer (1 M NaCl, 10 mM HEPES, pH 7.5, 1 mM EDTA, 0.5% Triton X-100). Before conducting SDS-PAGE analysis, equal amount of protein level were adjusted with 2X lysis buffer (0.125 M HCl, pH 6.8, 4% SDS, 20% glycerol, 2% 2-meracptoethanol and 0.02% bromophenol blue). The membranes were incubated with anti-Bcl2, Bax, cleaved- PARP and cleaved-caspase 3/9 (CST, Danvers, MA) at 1: 1000 dilutions, respectively, and anti-β-actin (Abcam, Cambridge, MA) at 1: 10,000 dilutions were kept for over 12 h at cold room. Horseradish peroxidase (HRP)-conjugated secondary antibodies were employed at 1: 5000 dilutions for 3 h at room temperature, the immune-complexes were visualized by the enhanced ECL method (Amersham Biosciences, Marlborough, MA).

### Caspase-3/9 activity assay

All processes were instructed following the protocol of caspase-3/9 assay kit (Biovision, Milpitas, CA). Olympus Fluoview FV1000 laser scanning confocal system (Olympus America Inc., Center Valley, PA) at 400 nm excitation and 505 nm emission was employed here for photosensitive reaction measurement. The fold of changes was calculated relative to the untreated cells.

### PI and Annexin V assay

Cells were plated and cultured in 24-well plates. After drug treatment for 48 h and labeled with Annexin V-FITC/PI apoptosis detection kit (BD Biosciences, Franklin Lakes, NJ) as followed manufacturer’s instructions. In brief, 100 μL of binding buffer containing annexin-V/FITC and propidium iodide (PI) were used for covering cells for 15 min at room temperature in the dark condition and then detected by the Olympus Fluoview FV1000 laser scanning confocal system.

### Statistical analysis and other assays

Protein levels were measured by Bradford’s method (Herculues, CA). Statistical tests have been done by using one-way analysis of variance. Data were expressed as Mean ± SEM, where *n* = 3–4. Statistically significant changes were classified as significant (*) where *p* < 0.05, more significant (**) where *p* < 0.01 and highly significant (***) where *p* < 0.001 as compared with control group. Statistically significant changes were classified as significant (^) where *p* < 0.05, more significant (^^) where *p* < 0.01 and highly significant (^^^) where *p* < 0.001 as compared with Aβ group.

## Results

### DBT attenuates Aβ-induced cell death

**Fig. 1:**
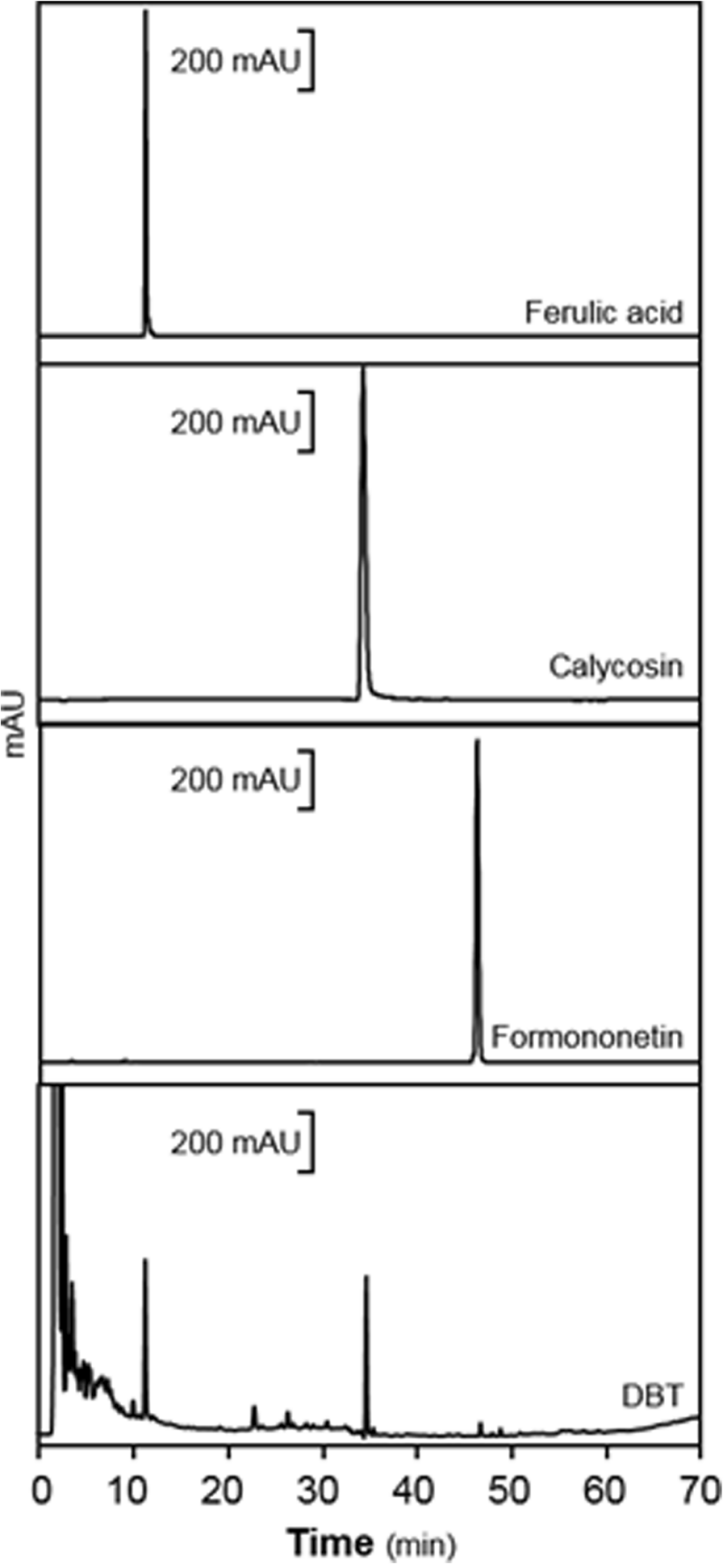
Typical HPLC fingerprint of DBT herbal extract. Typical chromatograph of major chemical markers and 1 mg/mL of DBT were subjected to HPLC analysis, and then a typical chromatograph was presented at a wavelength 254 nm

**Fig. 2:**
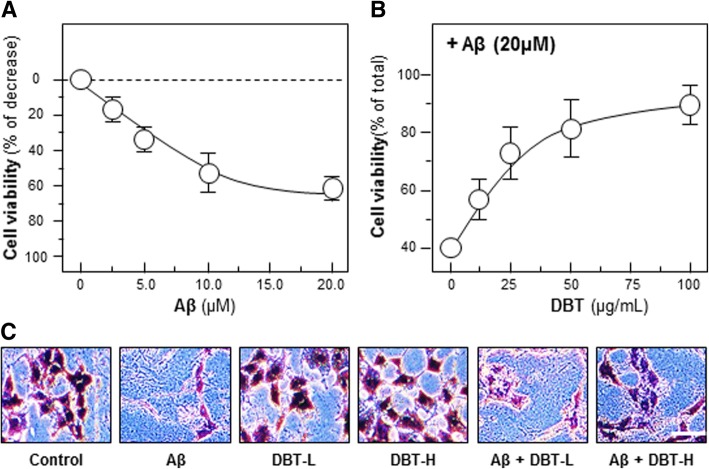
Neuroprotective functions of DBT against Aβ-induced cell death. **a** Cultured cortical neurons were treated with Aβ at different concentration for 48 h. **b** DBT extract (0–100 μg/mL) was applied 3 h before Aβ (20 μM) for another 48 h. **c** Crystal violet was employed for revealing cell viability after drug treatment for 48 h. Live cells were dyed and shown in purple color. In (**c**) and (**d**), Aβ (20 μM), DBT-L (12.5 μg/mL), DBT-H (100 μg/mL) were used here, and the treatment was similar as in (**b**). Values were expressed as the percentage of decrease or control reading in Mean ± SEM, where *n* = 3. Bar = 100 μm

**Fig. 3:**
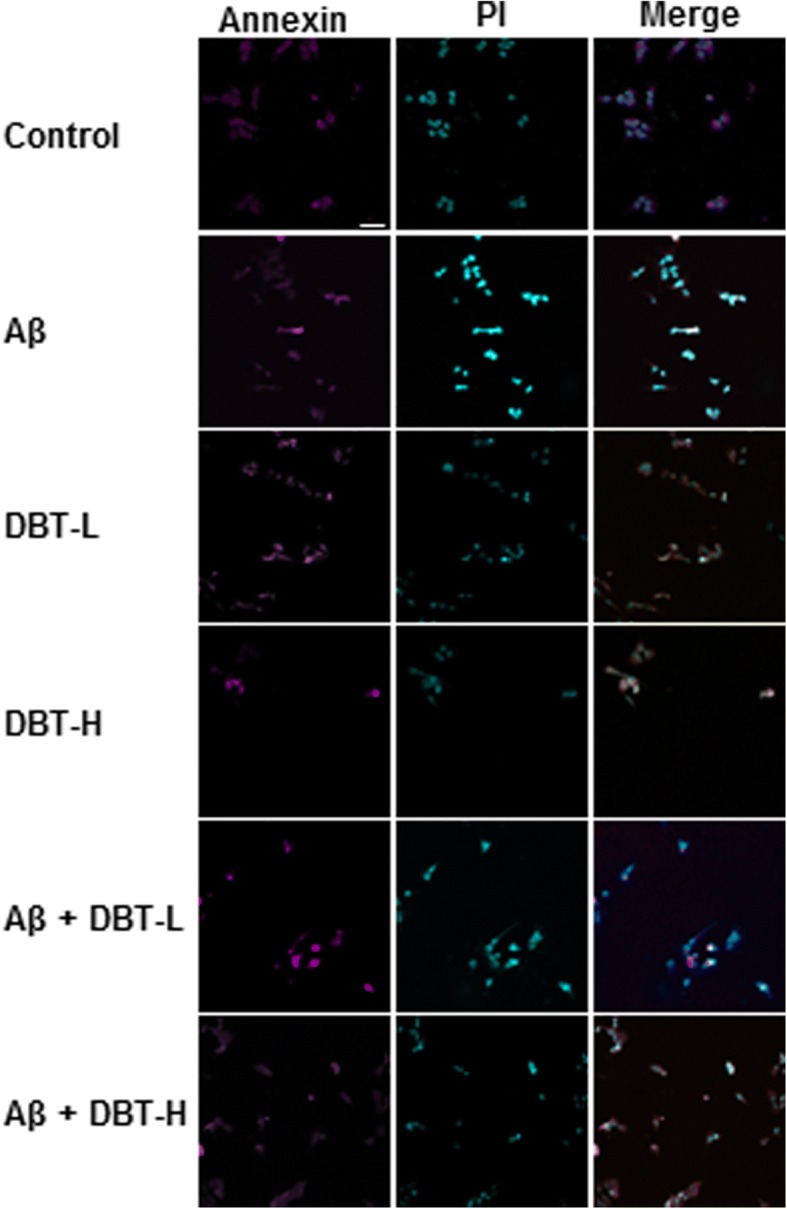
DBT inhibits Annexin V and PI rates. Representative images of Annexin V-FITC and PI fluorescence staining were shown for the cell apoptosis after drug treatment for 48 h (Aβ: 20 μM; DBT-L: 12.5 μg/mL; DBT-H: 100 μg/mL). Annexin V was visualized by purple signal, and PI was shown in blue color. Scale bar = 50 μM

Prior to the biochemical analysis of any herbal extract, it is indispensable to perform chemical composition analysis. The HPLC chromatogram of DBT water extract was shown here (Fig. 1). The accurate amounts of each major chemicals were calibrated by a calibration curve, which was obtained from a series of different concentration of the chemical markers. The calibration curve of ferulic acid was y = 21.134x + 19.607; calycosin was y = 10.189x-10.129; formononetin was y = 13.602x + 12.705. After calibrated by the standard curve, 1 g qualified DBT water extract contained 809.44 μg of ferulic acid, 693.19 μg of calycosin and 164.58 μg of formononetin. The cyto-protective functions of DBT against the Aβ-induced cell death in primary cultured neurons were analyzed. Application of Aβ in cultured cortical neurons provoked cell death obviously in a dose-dependent manner (Fig. 2a). From the results, the death rate was ~ 60% under the challenging of 20 μM of Aβ (Fig. 2a). The treatment of DBT reduced the Aβ-induced cell death in a concentration-dependent manner (Fig. 2b). Higher dose of DBT significantly protected cells from the injury: the maximum protection was able to recover cell number up to over 80% at 0.1 mg/mL (Fig. 2b). We also utilized crystal violet to determine cell viability, directly. The death cells lose their adherence, and finally resulted in reducing amount of crystal violet staining in cultures [23]. The DBT-treated cells remained healthy even after the challenge of Aβ (Fig. 2c). The sensitive apoptotic markers, Annexin V- and PI, were measured by fluorescent microscope. The Aβ-treated cells had a higher apoptotic rate of PI and Annexin V, as compared to the blank (Fig. 3). The ratio of Annexin V(+)/PI(+) of blank was 19.5%, positive treatment was 43.7%, lower concentration of DBT application (DBT-L: 12.5 μg/mL) was 19.6%, higher concentration of DBT challenging (DBT-H: 100 μg/mL) was 11.4%, the cotreatment of herbal decoction and Aβ were 41.6 and 21.8%, respectively (Fig. 3). The treatment of DBT suppressed the Aβ-induced cell apoptosis, dramatically (Fig. 3).

The aforementioned results showed that DBT could protect cell death against Aβ challenge.

### DBT protects cells from apoptosis

**Fig. 4:**
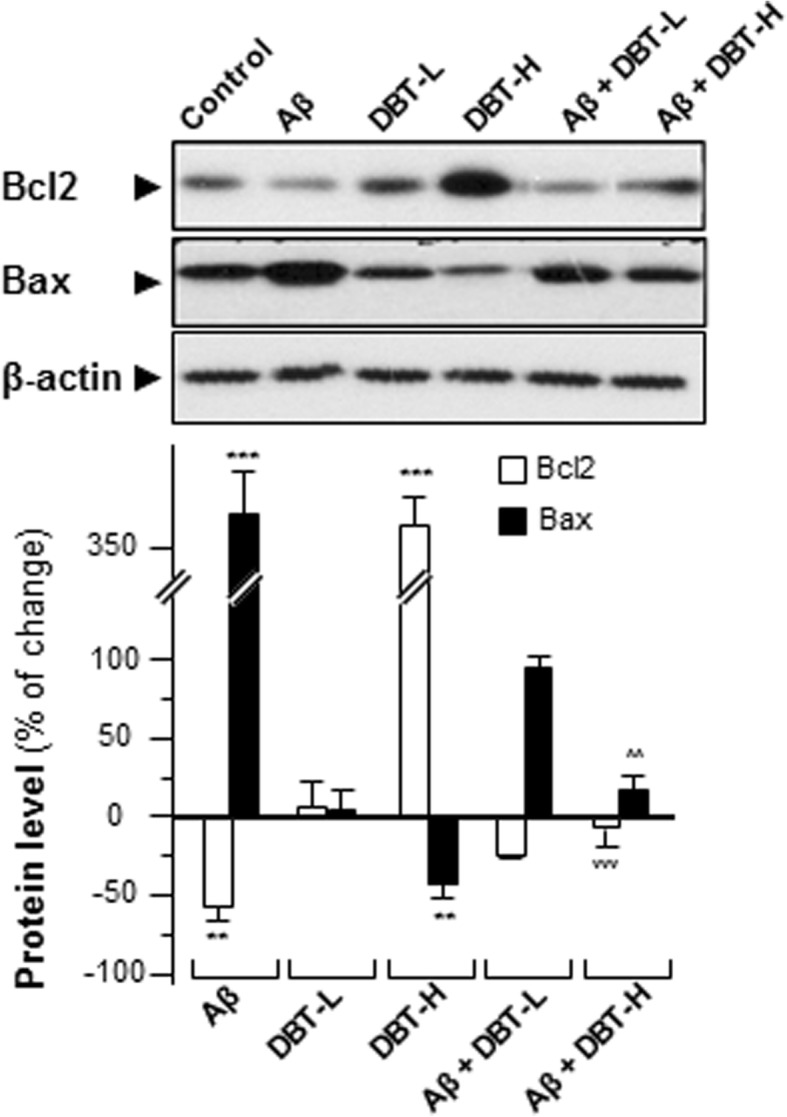
DBT regulates expressions of Bcl2 and Bax. Cultured cortical neurons were co-treated with various concentration of DBT (DBT-L: 12.5 μg/mL; DBT-H: 100 μg/mL) and Aβ (20 μM) for 48 h. The protein expression levels of Bcl2 (~ 27 kDa) and Bax (~ 21 kDa) were detected by immunoblot analysis using specific antibodies, and β-actin (~ 42 kDa) served as an internal control. Quantification of target protein expression was calculated by a densitometer. Values were expressed as the percentage of change as compared to control reading, in Mean ± SEM, where *n* = 3. Statistically significant changes were classified as more significant (**) where *p* < 0.01 and highly significant (***) where *p* < 0.001 as compared with control group. More significant (^^) where *p* < 0.01 and highly significant (^^^) where *p* < 0.001 are compared with Aβ group

Apoptosis is a programmed cell death, and inhibitors of apoptosis, e.g. Bcl2, and inducers of apoptosis, e.g. Bax, have been reported and identified [24]. The protein ratio of Bcl2 to Bax gives an index to the cell survival or death, after an apoptotic stimulus [25]. Here, we utilized western blot to reveal the protein expressions of two apoptotic markers in cultured cortical neurons, i.e. Bcl2 (~ 27 kDa) and Bax (~ 21 kDa) (Fig. 4). The application of Aβ significantly up-regulated the expression of Bax by over 3-fold and down-regulated Bcl2 by over 50%, as compared to the negative control (Fig. 4). Thus, Aβ markedly increased the ratio of Bax/Bcl2. Application of DBT in the cultures was capable to reverse the expressions of Aβ-induced cell apoptotic markers in a concentration-dependent manner (Fig. 4). More importantly, DBT decreased the ratio of Bax/Bcl2 obviously, as compared to the Aβ-treated group (Fig. 4), which suggested that this herbal decoction possessed neuroprotective effects in AD cell model.

**Fig. 5:**
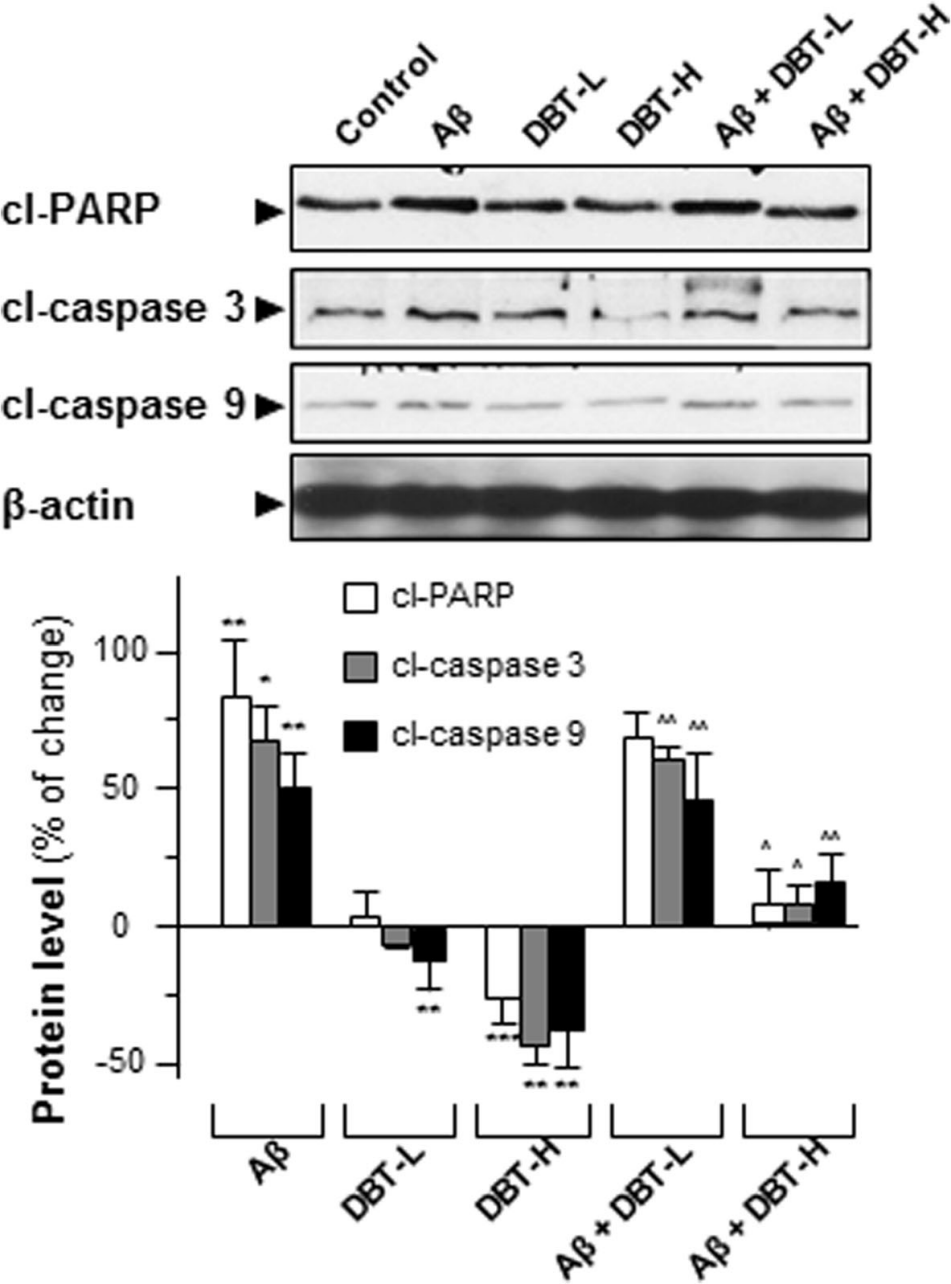
DBT attenuates the Aβ-induced apoptosis markers. Cultured cortical neurons were co-treated with different amounts of DBT (DBT-L: 12.5 μg/mL; DBT-H: 100 μg/mL) and Aβ (20 μM) or directly treated with Aβ or DBT alone for 2 days. The translational levels of cleaved-PARP (cl-PARP at ~ 85 kDa), cleaved-caspase 3 (cl-caspase 3 at ~ 17 kDa) and cleaved-caspase 9 (cl-caspase 9 at ~ 37 kDa) were detected by immunoblot analysis by specific antibodies. Beta-actin (~ 42 kDa) served as an internal control. Quantification of target protein expression was calculated by a densitometer. Values were expressed as the percentage of change as compared to control reading, in Mean ± SEM, where *n* = 3. Statistically significant changes were classified as significant (*) where *p* < 0.05, more significant (**) where *p* < 0.01 and highly significant (***) where *p* < 0.001 as compared with control group. Significant (^) where *p* < 0.05 and more significant (^^) where *p* < 0.005 are compared with Aβ group

**Fig. 6:**
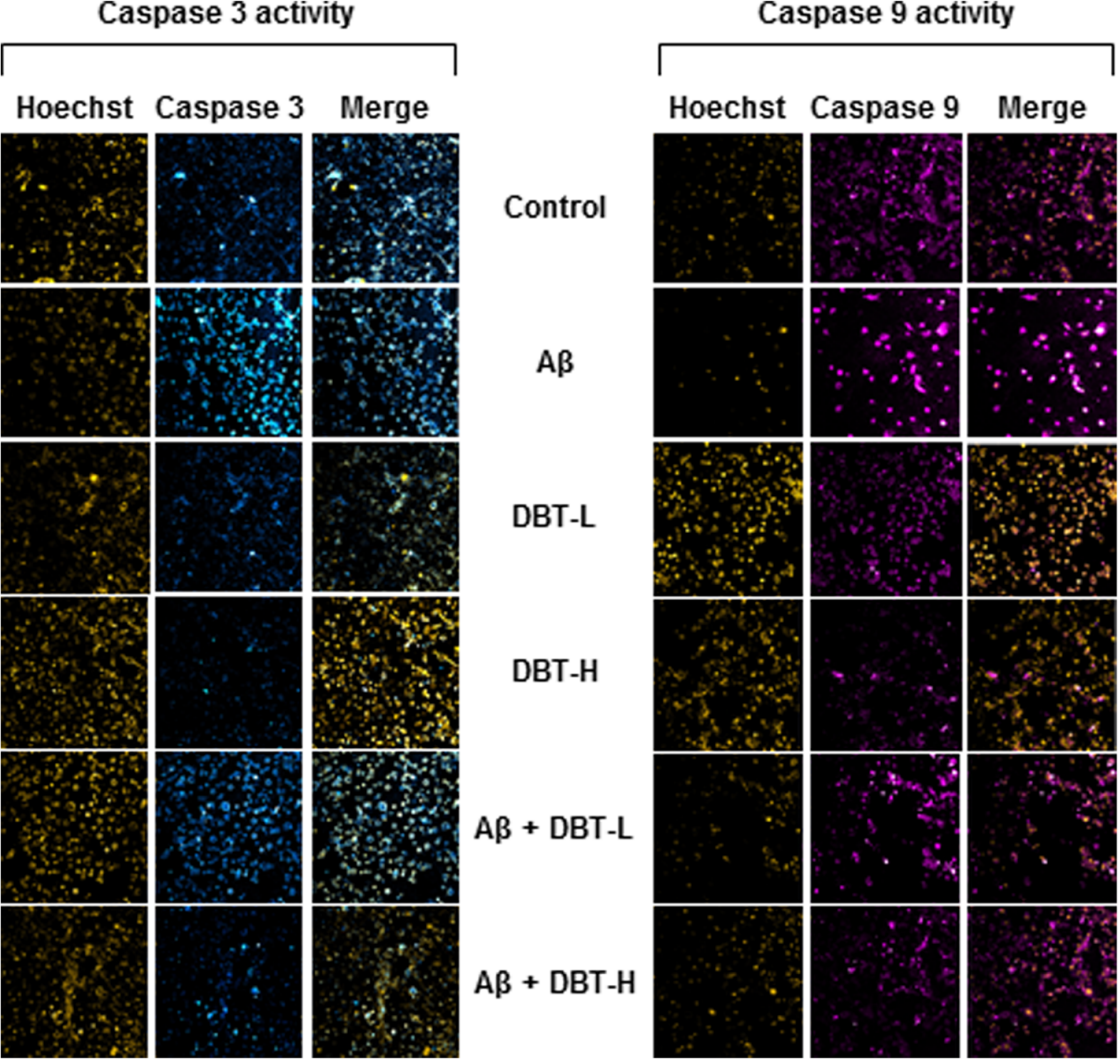
The treatment of DBT mitigates Aβ-triggered caspase 3/9 levels. Cultured cortical neurons were co-treated with a series amount of DBT (DBT-L: 12.5 μg/mL; DBT-H: 100 μg/mL) and Aβ (20 μM) for 48 h. Then cells were labeled with caspase 3/9 detection kit and Hoechst for 30 min. The activities of caspase 3/9 were evaluated by measuring the fluorescence intensity. Micrographs were taken by a confocal microscope

Caspases are a group of protease enzymes acting as important roles in manipulating cell apoptosis [26]. For example, the activation of caspases triggers degradation of cellular components in a programmed manner, which results in cell death [26]. In cultured cortical neurons, the amounts of cleaved-caspases 3, 9 and PARP were much higher in the Aβ-treated group, as compared to the negative control (Fig. 5). Challenging of DBT, on the other hand, suppressed the expressions of these cleaved-caspases in concentration-dependent manners (Fig. 5). The presence of high dose of DBT (0.1 mg/mL) in cultured cortical neurons decreased the levels of cleaved-PARP, caspase 3/9 at ~ 25%, ~ 50% and ~ 40% respectively, indicating the neuroprotective functions of DBT decoction (Fig. 5). Fluorescence signal, directly reflecting the amount of caspase activity in the cells, was also utilized and measured here [27]. In cultured cortical neurons, we found that the activities of caspases 3 and 9 were strongly activated in Aβ-treated group, as compared to others (Fig. 6). However, lower fluorescent signals were observed in DBT-treated group, and these results were fully in line with the western blotting results.

### Synergistic effects of AR and ASR

**Fig. 7:**
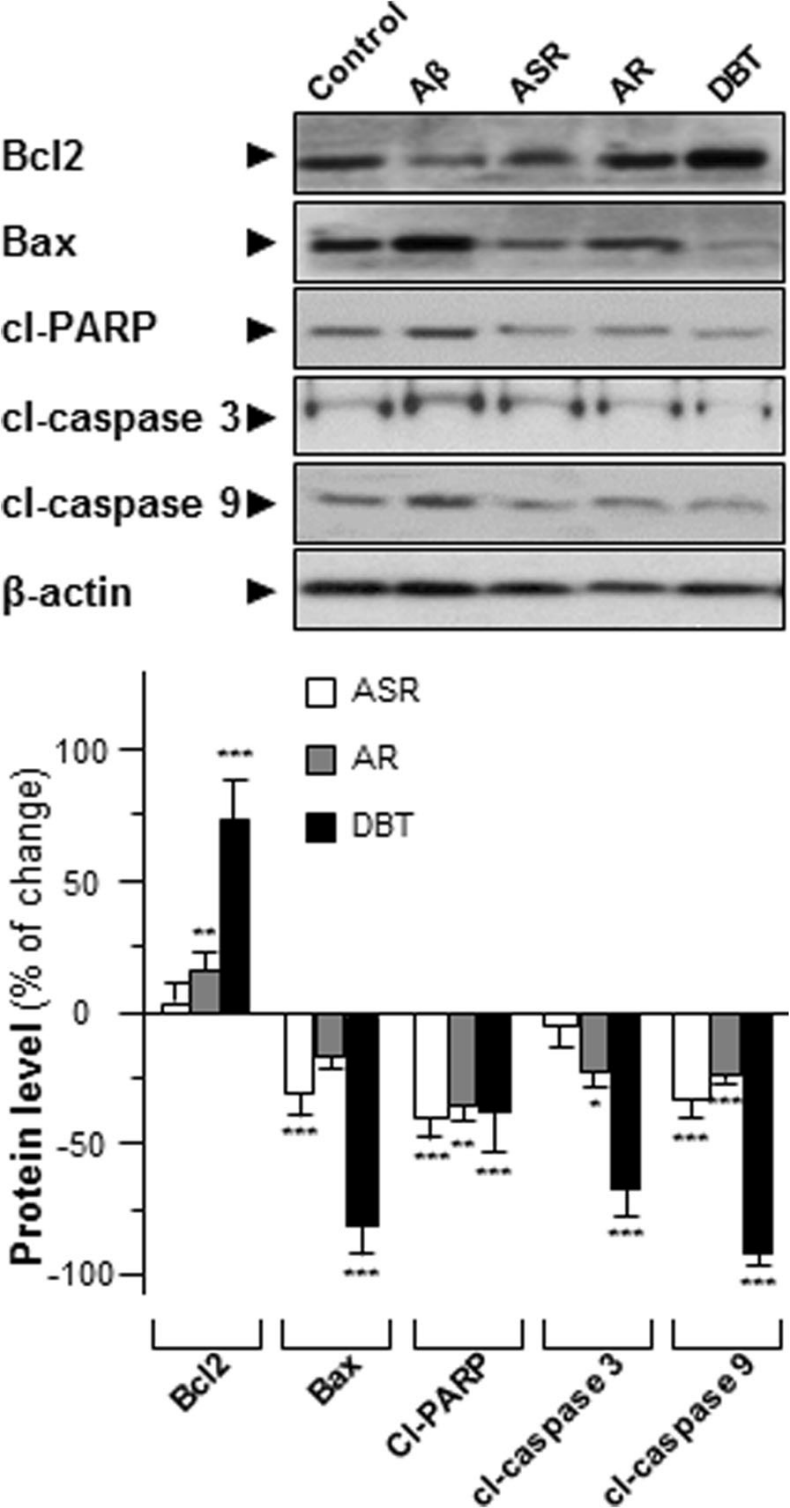
Synergistic properties of DBT extract. DBT (0.1 mg/mL), AR extract (0.1 mg/mL), ASR extract (0.1 mg/mL) were applied onto primary cultures for 2 days. The protein expressions of Bax (~ 21 kDa), Bcl2 (~ 27 kDa), cl-PARP (~ 85 kDa), cl-caspase 3 (~ 17 kDa) and cl-caspase 9 (~ 37 kDa) were detected by specific antibodies. Quantification of target protein expression was calculated by a densitometer. Values were expressed as the percentage of change as compared to control (untreated culture) reading, in Mean ± SEM, where *n* = 3. Statistically significant changes were classified as significant (*) where *p* < 0.05, more significant (**) where *p* < 0.01 and highly significant (***) where *p* < 0.001 as compared with control group

To reveal that synergistic effect of AR and ASR in the pharmacological properties of DBT, the specific apoptosis markers were determined here. One hundred μg of ASR significantly inhibited the expressions of Bax, cl-PARP and cl-caspase 9 in cultured neurons, as compared to the control. On the other hand, 100 μg of AR was capable to suppress the expressions of cl-PARP, cl-caspase 3/9 activities (Fig. 7). However, the application of DBT dramatically declined Bax/Bcl2 ratio and other apoptosis marker expression levels, and the rate of decline was much stronger than the sum of single application of ASR and AR (Fig. 7).

## Discussion

It is estimated that each year about 200,000 new cases of people younger than 65 years are suffering from AD, and the number is increasing, suggesting the younger onset of AD population. One of the targets for AD treatment is to prevent neuronal cell death. Being a neuroprotective agent, estrogen and its similar chemicals have drawn an attention, and they have been proven to enhance cognitive impairments both in vitro and in vivo [28]. Flavonoids, sharing the basic chemical structures with estrogen, also called phytoestrogen, are proposed to have neuroprotection, and which therefore are believed to pave a direction for new drug development [19, 28, 29]. In line to this notion, DBT contains high amounts of flavonoids, and theses flavonoids are classified as phytoestrogens [18, 19, 29]. Indeed, the flavonoids isolated from AR, i.e. calycosin and/or formononetin, are believed as an imperative and practical component found within this ancient herbal decoction [18]. To exert their biological functions in brain, flavonoids could cross the blood-brain barrier (BBB) before being delivered to the brain. BBB selectively restricts the passage of vast majority of small polar molecules and macromolecules from circulatory system to the brain [30]. Flavonoids, e.g. hesperetin, naringenin and their glucuronidated conjugates, have been shown to have good permeability across the BBB in vitro [31]. The epicatechin glucuronide was found in rat brain tissue after oral administration of epicatechin [32]. Collectively, the possible BBB permeable flavonoids are promising candidates for neuroprotective drug development. Indeed, the glucuronidated flavonoids are found abundantly in DBT [16, 17]. However, the therapeutic effect of flavonoids, so far, is very limited, and longtime consumption of flavonoid has been reported to trigger mutation and generation of free radicals [33].

The working mechanism of Aβ-induced neuronal cell death remains controversial. Free radical’s production and oxidative stress have been proposed to be ones of the causes. It is proposed that the Aβ-induced neurotoxicity is triggered by destabilization of intracellular Ca^2+^ and/or oxidative stress [2]. The apoptotic marker, Bcl-2, protects neurons against oxidative stress directly, and which maintains cellular Ca^2+^ hemostasis [34]. Therefore, Bcl-2 and the related anti-death proteins are acting as key regulators in monitoring programmed cell death in neurons. Therefore, the disruptions of these proteins could lead to an increased neuronal vulnerability. Here, we have shown the neuroprotection of DBT against Aβ-induced cell death in cultured cortical neurons. The treatment of this ancient herbal decoction in cultures could dramatically suppress the amounts of Bax and cleaved-PARP, cleaved-caspase 3/9, as induced by Aβ, indicating that the DBT was capable of attenuating Aβ-induced apoptosis. Moreover, the inhibitions of apoptotic markers, triggered by DBT, were much stronger than its herbal ingredients, suggesting that the boiling two herbs together at the ratio of 5:1 should be required. Furthermore, the in vivo data indicated that the formulation of AR and ASR at a 5:1 ratio was the most effective herbal decoction in triggering immune responses, enhancing formation of capillaries, up-regulating myocardial mitochondrial functions, as compared to AR or ASR herbal extract [17]. Higher pharmacological values of DBT than its ingredients could be explained by several possibilities. First, the AR-derived active components, i.e. saponins, may be able to enhance the pharmacological properties of active components from ASR. Second, the stability of active components could be prolonged by having a cocktail of DBT decoction [17]. Nevertheless, the pharmacological effects and the solubilities of active components within DBT are much stronger than that from AR or ASR extract.

The AR-derived calycosin, the most abundant flavonoid found within DBT decoction, was shown to induce nerve growth factor, glial-derived neurotrophic factor and brain-derived neurotrophic factor in cultured cortical neurons, and these inductions were in time- and dose-dependent manners [27]. This gene activation could be revealed similarly by the treatment of DBT [27]. By employing specific inhibitor of estrogen receptor, the activation of estrogen receptor was reported to be responsible for the calycosin-induced neurotrophic factor expressions [27]. In parallel, the in vivo rat middle cerebral artery occlusion model indicated that the treatment of calycosin could induce neuroprotection by up-regulating endogenous antioxidant and stabilizing the cells’ membrane structures [35]. Besides, mechanistic studies have proposed that calycosin is the key player in DBT function, as to achieve the highest pharmaceutical values in terms of estrogenic, osteogenic, erythropoietic and cardiovascular functions [10]. On other hand, AR-derived formononetin, and other isoflavonoid are highly enriched in DBT, which could exhibit anti-oxidation functions by suppressing H_2_O_2_-induced ROS formation, reducing Aβ aggregation; activating estrogen receptor and Akt phosphorylation, as well as suppressing the ratio of Bax/Bcl-2 [16]. The above-mentioned evidences indicated that AR could be a valuable herb in DBT for possible neuroprotective activities.

Ferulic acid is an antioxidant naturally presented in ASR. Long-term administration of ferulic acid protected against cognitive impairment-induced learning and memory deficits in vivo. Ferulic acid prevented peroxyl radical-trigged cell death in hippocampus as well as suppressed protein oxidation in response to hydroxyl and peroxyl radical generators, lipid peroxidation and ROS formation [21]. Moreover, ferulic acid protected against the free radical-mediated conformational changes in synaptosomal membrane proteins [21]. On the other hand, administration of Z-ligustilide, the primary lipophilic component in ASR, could diminish mortality, neurobehavioral deficits, brain edema, BBB permeability and cerebral vasospasm in AD rat model [20]. In parallel, Z-ligustilide was able to reduce apoptotic cells in the brain injury site by down regulating cleaved-caspase 3 and p53 expressions [20]. These data therefore suggested that ASR might be effective in AD patients.

The importance of “Blood” sheds light on the treatment of AD or other cognitive impairment [3, 4]. Various cognitive impairments are associated with micronutrient, especially iodine and iron [7]. In rats, iron deficiency induces brain mitochondrial damage and other alterations [5]. On the other hand, erythropoiesis involves a close interaction of iron and EPO. Iron is the fuel to produce new red blood cells. In addition to induce erythroblast differentiation, EPO stimulates endothelial proliferation, increases the expression of synaptophysin, enlarges capillary density, and decreases Aβ level in vivo, suggesting that EPO possesses therapeutic functions in mitigating AD syndromes [8]. Interestingly, our previous studies have shown that DBT decoction stimulated EPO expression via the HIF pathway [9, 16, 36]. Up to now, we do not know how the ancestors make up the formulation of DBT. Recent investigations in neurodegenerative disease models have provided glimpses that DBT decoction may be a fruitful therapeutic strategy for mitigating AD syndromes. However, the effect of DBT in AD animal model should be done as to support the possible usage of DBT in AD treatment.

## Conclusion

In cortical neuron cultures, DBT was capable of reducing the rate of Aβ-induced apoptosis in a dose-dependent manner; the effective concentration was revealed to be at 100 μg/mL. In the Aβ-treated cortical neurons, the expression ratio of Bcl2 to Bax was altered by DBT in a concentration-dependent manner. In parallel, DBT application could markedly suppress the Aβ-induced expressions of apoptosis markers, i.e. cleaved-caspase 3/9 and PARP. Taken these results, DBT sheds light in protecting the brain against stress or insult, but animal study is needed for further confirmation.

## Abbreviations

AD: Alzheimer’s disease
AR: Astragali Radix
ASR: Angelicae Sinensis Radix
Aβ: β-Amyloid
BBB: Blood brain barrier
DBT: Danggui Buxue Tang
ECL: Enhanced chemiluminesence
EPO: Erythropoietin
HRP: Horseradish peroxidase
PBS: Phosphate-buffered saline
PI: Propidium Iodide
ROS: Reactive oxygen species

## References

[1] Forner S, Baglietto-Vargas D, Martini AC, Trujillo-Estrada L, LaFerla FM (2017). Synaptic impairment in Alzheimer’s disease: a dysregulated symphony. Trends Neurosci.

[2] Murphy MP, LeVine H (2010). Alzheimer’s disease and the β-amyloid peptide. J Alzheimers Dis.

[3] Mazza M, Marano G, Traversi G, Bria P, Mazza S (2011). Primary cerebral blood flow deficiency and Alzheimer's disease: shadows and lights. J Alzheimers Dis.

[4] Aung SK, Fay Hm Hobbs RF (2013). Traditional Chinese medicine as a basis for treating psychiatric disorders: a review of theory with illustrative cases. Med Acupunct.

[5] Flores KP, Blohowiak SE, Winzerling JJ, Georgieff MK, Kling PJ. The impact of erythropoietin and iron status on brain myelination in the newborn rat. J Neurosci Res. 2018. 10.1002/jnr.24243. 10.1002/jnr.24243 29696692

[6] Lee ST, Chu K, Park JE, Jung KH, Jeon D, Lim JY, Lee SK, Kim M, Roh JK (2012). Erythropoietin improves memory function with reducing endothelial dysfunction and amyloid-beta burden in Alzheimer's disease models. J Neurochem.

[7] Ignacio J-L (2014). Iron deficiency and cognitive functions. Neuropsychiatr Dis Treat.

[8] Faux NG, Rembach A, Wiley J, Ellis KA, Ames D, Fowler CJ, Martins RN, Pertile KK, Rumble RL, Trounson B, Masters CL (2014). An anemia of Alzheimer's disease. Mol Psychiatry.

[9] Zhang WL, Zheng KY, Zhu KY, Zhan JY, Bi CW, Chen JP, Du CY, Zhao KJ, Lau DT, Dong TT, Tsim KW (2012). Chemical and biological assessment of *Angelica* herbal decoction: comparison of different preparations during historical applications. Phytomedicine.

[10] Gong AG, Lau KM, Xu ML, Lin HQ, Dong TT, Zheng KY, Zhao KJ, Tsim KW (2016). The estrogenic properties of Danggui Buxue Tang, a Chinese herbal decoction, are triggered predominantly by calycosin in MCF-7 cells. J Ethnopharmacol.

[11] Wang HL, Zhou QH, Meng BX, Zhou XL, Zheng GQ. Astragaloside IV for experimental focal cerebral ischemia: preclinical evidence and possible mechanisms. Oxidative Med Cell Longev. 2017;8424326. 10.1155/2017/8424326. 10.1155/2017/8424326 PMC5337886 28303172

[12] Li M, Li H, Fang F, Deng X, Ma S (2017). Astragaloside IV attenuates cognitive impairments induced by transient cerebral ischemia and reperfusion in mice via anti-inflammatory mechanisms. Neurosci Lett.

[13] Li L, Hou X, Xu R, Liu C, Tu M (2017). Research review on the pharmacological effects of astragaloside IV. Fundam Clin Pharmacol.

[14] Chao WW, Lin BF (2011). Bioactivities of major constituents isolated from *Angelica sinensis* (Danggui). Chin Med.

[15] Kim MC, Lee GH, Kim SJ, Chung WS, Kim SS, Ko SG, Um JY (2012). Immune-enhancing effect of Danggwibohyeoltang, an extract from Astragali Radix and Angelicae Gigantis Radix, in vitro and in vivo. Immunopharmacol Immunotoxicol.

[16] Lin HQ, Gong AG, Wang HY, Duan R, Dong TT, Zhao KJ, Tsim KW (2017). Danggui Buxue Tang (Astragali Radix and Angelicae Sinensis Radix) for menopausal symptoms: a review. J Ethnopharmacol.

[17] Dong TT, Zhao KJ, Gao QT, Ji ZN, Zhu TT, Li J, Duan R, Cheung AW, Tsim KW (2006). Chemical and biological assessment of a Chinese herbal decoction containing Radix Astragali and Radix Angelicae Sinensis: determination of drug ratio in having optimized properties. J Agric Food Chem.

[18] Xu SL, Bi CW, Choi RC, Zhu KY, Miernisha A, Dong TT, Tsim KW. Flavonoids induce the synthesis and secretion of neurotrophic factors in cultured rat astrocytes: a signaling response mediated by estrogen receptor. Evid Based Complement Alternat Med. 2013;127075. 10.1155/2013/127075. 10.1155/2013/127075 PMC3708423 23878590

[19] Yu DH, Duan YL, Bao YM, Wei CL, An LJ (2005). Isoflavonoids from Astragalus mongholicus protect PC12 cells from toxicity induced by L-glutamate. J Ethnopharmacol.

[20] Chen D, Tang J, Khatibi NH, Zhu M, Li Y, Wang C, Jiang R, Tu L, Wang S (2011). Treatment with Z-ligustilide, a component of *Angelica sinensis*, reduces brain injury after a subarachnoid hemorrhage in rats. J Pharmacol Exp Ther.

[21] Kanski J, Aksenova M, Stoyanova A, Butterfield DA (2002). Ferulic acid antioxidant protection against hydroxyl and peroxyl radical oxidation in synaptosomal and neuronal cell culture systems in vitro: structure-activity studies. J Nutr Biochem.

[22] Yan L, Hu Q, Mak MS, Lou J, Xu SL, Bi CW, Zhu Y, Wang H, Dong TT, Tsim KW (2016). A Chinese herbal decoction, reformulated from Kai-Xin-san, relieves the depression-like symptoms in stressed rats and induces neurogenesis in cultured neurons. Sci Rep.

[23] Feoktistova M, Geserick P, Leverkus M. Crystal violet assay for determining viability of cultured cells. Cold Spring Harb Protoc. 2016;prot087379. 10.1101/pdb.prot087379. 10.1101/pdb.prot087379 27037069

[24] Misao J, Hayakawa Y, Ohno M, Kato S, Fujiwara T, Fujiwara H (1996). Expression of bcl-2 protein, an inhibitor of apoptosis, and Bax, an accelerator of apoptosis, in ventricular myocytes of human hearts with myocardial infarction. Circulation.

[25] Oltvai ZN, Milliman CL, Korsmeyer SJ (1993). Bcl-2 heterodimerizes in vivo with a conserved homolog, Bax, that accelerates programmed cell death. Cell.

[26] Li J, Yuan J (2008). Caspases in apoptosis and beyond. Oncogene.

[27] Gong AG, Wang HY, Dong TTX, Tsim KWK, Zheng YZ (2017). Danggui Buxue Tang, a simple Chinese formula containing Astragali Radix and Angelicae Sinensis Radix, stimulates the expressions of neurotrophic factors in cultured SH-SY5Y cells. Chin Med.

[28] Genazzani AR, Pluchino N, Luisi S, Luisi M (2007). Estrogen, cognition and female ageing. Hum Reprod Update.

[29] Zhu JT, Choi RC, Chu GK, Cheung AW, Gao QT, Li J, Jiang ZY, Dong TT, Tsim KW (2007). Flavonoids possess neuroprotective effects on cultured pheochromocytoma PC12 cells: a comparison of different flavonoids in activating estrogenic effect and in preventing beta-amyloid-induced cell death. J Agric Food Chem.

[30] Pardridge WM (2005). Molecular biology of the blood-brain barrier. Mol Biotechnol.

[31] Youdim KA, Shukitt-Hale B, Joseph JA (2004). Flavonoids and the brain: interactions at the blood-brain barrier and their physiological effects on the central nervous system. Free Radic Biol Med.

[32] Abd El Mohsen MM, Kuhnle G, Rechner AR, Schroeter H, Rose S, Jenner P, Rice-Evans CA (2002). Uptake and metabolism of epicatechin and its access to the brain after oral ingestion. Free Radic Biol Med.

[33] Skibola CF, Smith MT (2000). Potential health impacts of excessive flavonoid intake. Free Radic Biol Med.

[34] Veis DJ, Sorenson CM, Shutter JR, Korsmeyer SJ (1993). Bcl-2-deficient mice demonstrate fulminant lymphoid apoptosis, polycystic kidneys, and hypopigmented hair. Cell.

[35] Kumar S, Reddy PH (2016). Are circulating microRNAs peripheral biomarkers for Alzheimer's disease?. Biochim Biophys Acta.

[36] Zheng KY, Choi RC, Cheung AW, Guo AJ, Bi CW, Zhu KY, Fu Q, Du Y, Zhang WL, Zhan JY, Duan R, Lau DT, Dong TT, Tsim KW (2011). Flavonoids from Radix Astragali induce the expression of erythropoietin in cultured cells: a signaling mediated via the accumulation of hypoxia-inducible factor-1α. J Agric Food Chem.

